# Evaluation of antibody immunochromatography testing for diagnosis of current *Helicobacter pylori* infection

**DOI:** 10.1016/j.plabm.2021.e00245

**Published:** 2021-07-24

**Authors:** Jamil M.A.S. Obaid, Ayoob N.N. Ayoon, Omar N.M. Almurisy, Sammer M.S. Alshuaibi, Nashwan N. Alkhawlani

**Affiliations:** a Medical Laboratory Sciences Dept., Faculty of Medicine and Health Sciences, Ibb University, Yemen; b Medical Microbiology Dept., Faculty of Science, Ibb University, Yemen

**Keywords:** *H. pylori*, Gastric ulcer, Stool antigen test, Serum antibody test, Sensitivity, Specificity, Immunochromatography

## Abstract

**Background:**

*Helicobacter pylori* infection represents a major gastrointestinal complaint associated with gastritis, gastric ulcer and stomach tumors. It is present in 90 % of developing countries population. *H. pylori* diagnosis in these countries, where resources are limited, is accomplished with simple non-invasive tests such as stool antigen and serum antibody tests. The aim of this study was to evaluate the serum antibody test in the diagnosis of current *H. pylori* infection.

**Subject and methods:**

A total of 117 patients were included in this prospective diagnosis accuracy testing study, who clinically presented with dyspepsia, heartburn, abdominal pain, diarrhea, or halitosis. A stool sample was collected from each patient and tested for *H. pylori* antigen using immunochromatographic method.

Blood sample was also collected, half of which was EDTA-sampled and analyzed for complete blood count, while the remaining half was left to clot, the separated serum was tested for antibodies against *H. pylori* with immunochromatographic cassette.

**Results:**

About 35 % of sixty six patients who were positive for stool antigen test gave a negative for serum antibodies test. Meanwhile, the non-consistent results within 51 negative stool antigen test patients was exhibited by 47 % of them. The discrepancies were not affected by age or disease duration. The calculated sensitivity, specificity, positive predictive value and negative predictive values were 50 %, 65 %, 65 % and 50 % respectively.

**Conclusion:**

The serum antibody test is not reliable in the diagnosis of current *H. pylori* infection. In developing countries, with limited facilities and primary care units, stool antigen test diagnosis is useful for diagnosis.

## 1 Introduction

*Helicobacter pylori* is a major causative agent of gastritis, gastric ulcer and malignant tumors of the stomach; carcinoma and lymphoma [[1], [2], [3]]. The prevalence of *H. pylori* infection within the developed population saw about 40 %, however it reached to about 70 % within the population of the developing countries [4]. In Yemen Republic, the published studies state that the prevalence of *H. pylori* among symptomatic child patients was 65 % and 9 % among asymptomatic healthy Yemeni children [5,6]. Another study in Yemen showed that the prevalence of *H. pylori* infection within hospitalized patients, who had undergone upper gastrointestinal endoscopy is very high (98.7 %). This same study concluded that *H. pylori* is significantly associated with oesophagitis, gastritis and peptic ulcer in Yemen [7]. The high prevalence rate is attributed to the poor hygiene, sanitation problems and low socioeconomic state of people in the poorer countries, in addition to the problem of obtaining clean sources of water supply.

Fecal-oral, oral-oral, fly-mediated, waterborne and iatrogenic (through endoscopy) transmission modes of *H. pylori* have been reported [8,9]. Four steps are critical for *H. pylori's* colonization and pathogenesis; survival under acidic stomach conditions; movement toward epithelium cells through flagella-mediated motility; attaching to host receptors by adhesins and tissue damage by toxin release; this damage may develop to severe gastric lesions or peptic ulcer disease [10]. Infection with *H. pylori* carrying specific virulence factors can lead to different severe outcomes [11].

The adaptive immune response to *H. pylori* is characterized by proinflammatory response with Th1 and Th17 subsets which contributes to protection. This response also supports chronic inflammation and injury that can ultimately lead to development of gastric cancer [12]. The humoral immune response is minimum and indicated by low IL-4 or IL-5, and high IFN-γ production by CD4^+^ T cell clones of *H. pylori*-infected patients. Despite the detection of serum IgA and IgG antibodies in chronically infected persons, the IgA- and IgM-secreting cells in *H. pylori* infected patients were elevated in contrast to IgG-secreting cells [13,14].

Patients with *H. pylori* infection are usually admitted to hospital with overt clinical symptoms like dyspepsia, heartburn, abdominal pain, diarrhea, or halitosis. The laboratory diagnosis can be made using samples collected by invasive and non-invasive methods. In developing countries with limited resources and low health facilities, diagnosis with low-cost non-invasive methods, such as antigen testing from stool sample and antibody testing from serum, are preferred. These tests are familiar in routine clinical practice in Yemen Republic. This paper evaluates the serum antibody testing with immunochromatography technique in comparison with stool antigen testing, for *H. pylori* infection's diagnosis.

## 2 Subjects and methods

### 2.1 Subjects

This prospective diagnosis accuracy testing study was conducted on 117 patients, 76 % of them were females and 24 % were males. All patients attended public private hospitals or outpatient clinics in Ibb city during the period between April and June 2019. A stool sample from each patient was tested for *H. pylori* antigen using immunochromatography technique. A blood sample was also collected, half of which was EDTA sampled and analyzed from complete blood count, while the remaining half was left to clot and the serum was separated to be used for *H. pylori* antibodies testing. An informed consent was taken from each patient before enrollment in this research.

### 2.2 Methods

#### 2.2.1 *H. pylori* stool antigen test by immunochromatography

A small piece of stool sample was prepared for testing by dissolving it in a buffer solution provided by the manufacturer (InTec products, INC. Haicang, Xiamen, China) and mixed thoroughly. After 3–10 min, three drops of mixture were put in the sample well of the immunochromatography cassette. The result was read after incubation for 10 min. Validity of the cassette was confirmed by monitoring the development of the precipitation line of the control on the strip.

#### 2.2.2 *H. pylori* serum antibody test by immunochromatography

Immunochromatography cassettes for antibody testing from the same manufacturer were used. One drop of serum sample was put in the testing orifice with two drops of the provided buffer and left to migrate for 10 min before the result was read. The validity of the cassettes was confirmed by monitoring the development of precipitation line in the control area on the strip.

#### 2.2.3 CBC

The EDTA blood sample that was collected from each patient was analyzed for complete blood count using a full automatic hematology analyzer (Sysmex xsi-500 Europe GmbH, Germany) with five part differential leukocytes parameters output.

#### 2.2.4 Statistical analyses

The quantitative data was presented with descriptive statistics and tested for difference with a simple *t*-test using IBM SPSS software version 19, (property of SPSS 2010, Inc., IBM Company).

## 3 Results

A total of 117 patients were included in this study with more propensity of female (76 %) and adults whose ages ranged from 31 to 60 years old. Most of the patients live in a low socioeconomic state, so most of them get their water requirements from non-refined water (79 %) and their eating routinely was at home (94 %) (Table 1).

**Table 1:** Patients’ demography.

Data	Groups	No.	Percentage
**Sex**			
	Female	89	76
	Male	28	24
**Age**			
	<15 y	9	8
	15-30 y	39	33
	31-60 y	62	53
	>60 y	7	6
**Water source**			
	Refined	25	21
	Tap	92	79
**Eating source**			
	Restaurant	7	6
	Home	100	94

The hematological variables did not statistically differ between the two patient groups; *H. pylori* antigen-positive and *H. pylori* antigen-negative as listed in Table 2. It was noted that the main neutrophil count was higher within antigen positive patients than those who were antigen negative despite the lack of statistical significance. In contrast, the mean lymphocyte count was higher within antigen negative patients than it was with the positive ones.

**Table 2:** Hematology parameters of antigen positive and antigen negative *H. pylori* patients.

Patient groups	Hb	WBC	PLTs	Neutrophils	Lymphocytes
	Mean ± SD	Mean ± SD	Mean ± SD	Mean ± SD	Mean ± SD
Antigen positive	13.1 ± 1.6	5.9 ± 1.9	324 ± 122	52.4 ± 15.7	38.1 ± 11.7
Antigen negative	16.3 ± 18.6	6.3 ± 2.4	301 ± 84	46.4 ± 16.2	40.1 ± 14.1
*p value*	0.205	0.633	0.633	0.097	0.494

Hb; Hemoglobin, WBC; White blood cells, PLTs; Platelets.

Among the positive stool antigen testing results about 35% showed a negative antibody result, with the mean age of 34.2 years. On the other hand, about 47% of the negative stool antigen testing results exhibited a positive antibody test result. The mean age of the participants was 33.5 years and 13% of them were males (Table 3, Table 4).

**Table 3:** Performance of antigen and antibody testing for diagnosis of current *H. pylori* infection.

Antigen testing	Antibody testing		Total
	Positive	Negative	
Positive	33	33	66
Negative	18	33	51
Total	51	66	117

**Table 4:** Performance of antigen and antibody testing for both sexes.

Sex	Antigen testing	Antibody testing		Total
		Positive	Negative	
Males	Positive	2	4	**6**
	Negative	6	16	**22**
	**Total**	**8**	**20**	**28**
Females	Positive	31	29	**60**
	Negative	12	17	**29**
	**Total**	**43**	**46**	**89**

To evaluate the serum testing for *anti*-*H. pylori* antibodies by immunochromatography in clinical practice for current *H. pylori* infections diagnosis, the evaluation analyses (sensitivity, specificity, positive predictive value (PPV) and the negative predictive value (NPV)) were calculated with reference to stool antigen testing performed with the same immunochromatography technique. The results of both tests on patients’ samples were cross tabulated in Table 3, Table 4 The calculated evaluation values indicate to a sensitivity of 50% and a specificity of 65%. Meanwhile, the PPV was 65% and the NPV was 50%.

The evaluation analyses for both sexes revealed an increase in sensitivity and PPV of females compared with that of males, who revealed an increase in specificity and NPV compared with females. The sensitivity, specificity, PPV and NPV for female patients were 52 %, 59 %, 72 % and 37 % respectively, while those of males were 33 %, 72 %, 25 % and 80 % respectively (Table 4).

## 4 Discussion

The high prevalence of *H. pylori* infection in developing countries may reach to 90 % of the populations [15] which makes it a major gastrointestinal problem in these countries. This health problem is wide spread with water contamination, poor hygiene, bad sanitation and low socioeconomic state of citizens. All these factors are constantly present in the developing countries, including Yemen Republic, and mean that this disease is endemic. The majority of patients eat their meals at home, which indicates to unhealthy living conditions.

In the developing countries, the available diagnostic tests of current *H. pylori* infection are the cheaper ones that are categorized as non-invasive tests, and these mainly depend on the simple immunochromatographic technique of both stool antigen and serum antibody testing. Immunochromatography is based on lateral flow chromatography with antigens or antibodies and then detection with golden beads. In clinical practices, the immunochromatographic-based test is more convenient for routine use in small clinics. Other non-invasive tests (such as urea breath test) and invasive methods (e.g. endoscopic biopsy) are highly expensive and rarely used. The commonly used diagnostic tests in about 99 % of the public and private medical laboratories in Yemen are the stool antigen immunochromatography test and the serum antibody immunochromatography test.

The stool antigen test was evaluated as a reliable diagnostic test for *H. pylori* by many studies, in comparison with another gold standard methods such as breath test and biopsy bacterial culture. It was recommended as a useful test for small laboratories that do not have advanced equipment [16]. Moreover, these studies that were carried out to evaluate the stool antigen test in comparison with gold standard methods concluded a sensitivity of 88.9 %–98.3 %, and a specificity of 77.8 %–98.4 % [[17], [18], [19]]; these values are non-consistent with our values of serum antibody testing.

Silva et al. in their study on the validation of stool antigen testing, inferred a sensitivity of 88 %, specificity 87.5 %, PPV 80 % and NPV 87.5 %. They concluded that the lateral flow stool antigen test can be used as an alternative to a breath test for diagnosis of primary infection of *H. pylori*, especially in developing countries [16].

Abadi A., in his review, lists the characteristics of stool antigen testing and reported high specificity (>95 %) and sensitivity (>95 %), in addition to its popularity among patients, being relatively fast and simple, and not requiring skilled staff [20]. The limiting factors of this test are upper gastrointestinal bleeding, antibiotic consumption, bowel movement, and proton pump inhibitors uptake [21]. Abadi A, states that there is a good agreement between published guidelines and consensuses on the stool antigen test using monoclonal antibodies to be regarded as one of the best approaches in the measurement of and successful eradication of the bacterium *H. pylori* and also for its primary detection in clinical settings [20].

Based on that data about antigen testing, the validation of serum antibody testing for *H. pylori* current infection was carried out in this study in reference to stool antigen testing. The serum antibody test exhibited a lower sensitivity and specificity in comparison with that obtained from a study in Japan [22]. This may be attributed to the difference in prevalence rate of infection between developed and developing countries, and in some aspects due to the quality of kits used. Therefore, it is possible that the serum antibody test that may be useful for diagnosis of current *H. pylori* infection in developed countries is less useful in developing countries.

The patient genders exhibited a difference in sensitivity, specificity, PPV and NPV values. This may be attributed to the behavioral difference in their life style, for example the males spend all their time out of doors and exposed to more contamination and consequently to the type and extent of immune response. The possibility for exclusion of infection in the negative serum antibody tests is increased if the patient is male because of better specificity. In contrast, the possibility to confirm *H. pylori* infection with a positive serum antibody test result increased if the patient was female due to better sensitivity. Finally, the gender difference might be inconclusive and needs more evidence accumulation to reach a conclusive comprehension about it.

The non-consistent stool antigen test result and serum antibody test result were not affected by age, where the mean ages of patients with different results are close (33.5 and 34.2 years), neither did it depend on the disease duration (19.8 and 19.4 months). This inconsistent of antigen and antibody test results have been reported previously [23]. The non-consistent results may be due to a difference in immune system capability of patients and seroconversion, past history of infection and other interfering substances in both tests. Nevertheless, the difference in mean neutrophils and lymphocytes count values -the tools of innate and adaptive immune response-within antigen-positive and antigen-negative groups may enhance the effect of immune system.

In conclusion, the serum antibody test cannot be used as an alternative test to the stool antigen test in detection of current *H. pylori* infections. The stool antigen test would be more useful than the serum antibody test in circumstances of limited resources and in all primary care units of developing countries.

## Declaration

This paper has no conflict of interest and is the responsibility of authors.

## Consent for publication

The authors give consent for publication to Practical Laboratory Medicine.

## Availability of data and materials

All data are available in this manuscript.

## Funding

The authors did not receive any funding.

## Authors' contributions

JO contributed in study planning, writing, analyses and preparing the paper for publication. All others contributed in sample collection and lab analyses. All authors have read and approved the manuscript.

## Declaration of competing interest

This paper has no conflict of interest and a responsibility of authors.
